# 15 kDa Granulysin versus GM-CSF for monocytes differentiation: analogies and differences at the transcriptome level

**DOI:** 10.1186/1479-5876-9-41

**Published:** 2011-04-18

**Authors:** Luciano Castiello, David F Stroncek, Michael W Finn, Ena Wang, Francesco M Marincola, Carol Clayberger, Alan M Krensky, Marianna Sabatino

**Affiliations:** 1 Cell Processing Section, Department of Transfusion Medicine, Clinical Center, National Institutes of Health, Bethesda, MD 20892, USA; 2 Laboratory of Cellular and Molecular Biology, National Cancer Institute, National Institutes of Health, Bethesda, MD 20892, USA; 3 Infectious Disease and Immunogenetics Section, Department of Transfusion Medicine, Clinical Center, and Center for Human Immunology (CHI), National Institutes of Health, Bethesda, MD 20892, USA

## Abstract

**Background:**

Granulysin is an antimicrobial and proinflammatory protein with several isoforms. While the 9 kDa isoform is a well described cytolytic molecule with pro-inflammatory activity, the functions of the 15 kDa isoform is less well understood. Recently it was shown that 15 kDa Granulysin can act as an alarmin that is able to activate monocytes and immature dendritic cells. Granulocyte Macrophage Colony Stimulating Factor (GM-CSF) is a growth factor widely used in immunotherapy both for *in vivo *and *ex vivo *applications, especially for its proliferative effects.

**Methods:**

We analyzed gene expression profiles of monocytes cultured with 15 kDa Granulysin or GM-CSF for 4, 12, 24 and 48 hours to unravel both similarities and differences between the effects of these stimulators.

**Results:**

The analysis revealed a common signature induced by both factors at each time point, but over time, a more specific signature for each factor became evident. At all time points, 15 kDa Granulysin induced immune response, chemotaxis and cell adhesion genes. In addition, only 15 kDa Granulsyin induced the activation of pathways related to fundamental dendritic cell functions, such as co-stimulation of T-cell activation and Th1 development. GM-CSF specifically down-regulated genes related to cell cycle arrest and the immune response. More specifically, cytokine production, lymphocyte mediated immunity and humoral immune response were down-regulated at late time points.

**Conclusion:**

This study provides important insights on the effects of a novel agent, 15 kDa granulysin, that holds promise for therapeutic applications aimed at the activation of the immune response.

## Background

Many immunotherapies are based on the use of immunomodulators for the activation or suppression of the immune response. These immunomodulators include cytokines, chemokines and growth factors that act on specific subsets of immune cells *in vivo *or *ex vivo*, alone or in combination, to modulate an immune response.

GM-CSF is a growth factor encoded by the CSF2 gene[1]. It is a glycoprotein naturally produced by lymphocytes and monocytes that induces the *ex vivo *proliferation of hematopoietic progenitor cells to form colonies of mature blood cells[2]. In addition, GM-CSF induces the proliferation of monocytes-macrophages and secretion of inflammatory cytokines such as tumor necrosis factor (TNF) and interleukin 1 (IL-1)[3]. It plays an important role in the activation of dendritic cells (DCs), T cells and natural killer (NK) cells[2]. Because of its role in modulating both the innate and adaptive immune responses, GM-CSF has been used for immunotherapies both *in vivo *and *ex vivo. In vivo *alone and in combination with other cytokines, it enhances antigen presentation of cancer cells [4,5] and stimulates autologous immune responses [1,2]. It has also been used as a tumor vaccine adjuvant[1]. *Ex vivo *applications of GM-CSF are mainly related to the differentiation of monocytes into immature DCs in combination with IL-4 [6], IL-15 [7], interferon α (IFN- α) [8], or as a single agent [9]. At a molecular level, GM-CSF induces monocyte expression of IL-10 [10], IL-3R [11], CD23 (FCER2) [12], CD1 [13] and regulates the expression of MHC class II antigens [14]. However, the molecular effects of GM-CSF on monocytes *in vitro *have not yet been completely characterized.

Granulysin is a member of the saposin-like protein (SAPLIP) family[15] and colocalizes in the granular compartments of human cytotoxic T lymphocytes (CTL) and NK cells along with granzymes and perforin [16]. It is encoded by GNLY and is a glycoprotein with at least 4 different isoforms[15]. The "mature" granulysin protein (9 kDa) results from the proteolytic maturation of a "secretory" 15 kDa precursor. The 9 kDa isoform is a well characterized proinflammatory cytokine with cytolitic activity[17]. It is able to induce cytolysis of various types of tumors and microbes and induces the expression of several cytokines, such as CCL5 (RANTES), CCL2 (MCP1), CCL4 (MIP-1β), IFNα, and IL-1[17]. The 15 kDa protein is constitutively secreted but its physiological roles have only recently been elucidated [18]. Several diseases, including infections, cancer, autoimmune and skin ailments, are characterized by an abnormal level of expression of Granulysin, suggesting a possible role in regulating immune response and the normal physiology[17]. Recently it has been shown that both 9 and 15 kDa recombinant Granulysin are able to activate antigen presenting cells and act as immune alarmins [18]. In fact, they induced in vitro chemotaxis and activation of both human and mice DCs and inflammatory leukocytes[18]. Of note, 15 kDa Granulysin is much more potent in chemotaxis and proinflammatory activities than the 9 kDa isoform [18] and while the 9 kDa isoform is a potent antimicrobial and tumoricidal agent, the 15 kDa form has no cytolytic activity *in vitro *(Clayberger *et al.*, submitted for publication).

In the present study, we performed gene expression analysis of monocytes cultured for 4, 12, 24 and 48 hours in presence of either GM-CSF or 15 kDa Granulysin. This analysis showed that a common signature could be identified at each time point, but over time, different specific effects could be assigned to each of the cytokines relevant to monocyte differentiation and potential therapeutic use. In particular, GM-CSF specifically modulated the expression of several genes involved in the cell differentiation, whereas Granulysin specifically induced the expression of proinflammatory cytokines.

## Methods

### 15 kDa Granulysin expression and purification

A detailed description of the procedure has been previously described by Finn et al, 2011[19]. Briefly, a cDNA clone of the 15 kDa Granulysin gene was generated from human peripheral blood cells and cloned into a pet28A *E. coli *expression vector. After being engineered for insect expression and secretion, the vector was transfected in Hi5 insect cells and after 2 days of culture at 21 C the supernatant was filtered using a 0.45 μM filter and applied to a 5 ml HiTrap Heparin HP (GE Health Care, Uppsala, Sweden). Fractions containing the 15 kDa Granulysin were pooled, purified on 1 ml Resource S column (GE Health Care), concentrated and stored at -80°C.

### Cell Culture

Human peripheral blood from three healthy donors was collected by apheresis in the Department of Transfusion Medicine of the Clinical Center (NIH) using Amicus Separator (Baxter Healthcare Corp., Fenwal Division, Deerfield, IL). The monocyte fraction was immediately separated by elutriation (Elutra^®^, Gambro BCT, Lakewood, CO, USA) according to the manufacturer's instructions and the purity achieved was greater than 80%. Fresh monocytes were cultured in 6-well plates (Corning Costar, Corning Incorporated, Corning, NY, USA) at a concentration of 2 ×10 ^6 ^cell/ml in 90% RPMI-1640 media, 10% AB heat inactivated plasma, 10 mcg/ml gentamicin in the presence of 15 kDa Granulysin (10 nM) or GM-CSF (Leukine Sagramostin, 10 ng/ml, 56 IU/ml, Genzyme, Cambridge, MA, USA) and harvested at 4, 12, 24 and 48 hours.

### RNA extraction

At times 0, 4 h, 12 h, 24 h and 48 h 20 ×10 ^6 ^cells from each culture condition were used for total RNA extraction using miRNA Easy Kits (Qiagen, Valencia, CA, USA). RNA quantity and quality were assessed by ND-1000 Spectrophotometer (NanoDrop Technologies, Wilmington, DE, USA) and Agilent 2100 Bioanalyser (Agilent Technologies, Waldbronn, Germany), respectively.

### Microarray Analysis

Samples and universal Human Reference RNA (Stratagene, Santa Clara, CA, USA) were amplified and labeled using Agilent kit according to the manufacturer's instructions and hybridized on Agilent Chip (Whole Human genome, 4 × 44 k, Agilent Technologies, Santa Clara, CA, USA). The arrays were scanned with Agilent Microarray Scanner and the images were analyzed using Agilent Feature Extraction Software 9.5.1.1. Resulting data were uploaded onto mAdb Gateway http://madb.nci.nih.gov, retrieved and analyzed with BRB Array Tools http://linus.nci.nih.gov/BRB-ArrayTools.html. The raw data set was filtered according to a standard procedure to exclude spots below a minimum intensity of 20 in both fluorescence channels. If the fluorescence intensity of one channel was higher than 20, but the other was below 20, the fluorescence of the low intensity channel was arbitrarily set to 20. Flagged spots were also excluded from the analysis. A total of 33757 genes passed the filter and were used for the analysis.

### Real Time PCR Analysis

A total of 0.5 μg of purified RNA was used to synthesize cDNA using Random Hexamers (Qiagen, Valencia, CA, USA) and Superscript II RT (Invitrogen, Carlsbad, CA, USA) according to the manufacturer's instruction. The expression of CCL2, CCR7, CD209 and PIM1 were tested using specific TaqMan Gene Expression Assays (Applied Biosystems, Carlsbad, CA, USA). HPRT1 was selected as the housekeeping gene, due to the fact that it has been described as a housekeeping gene in monocytes [20] and it showed low variability in our microarray dataset. RT-PCR reactions were setup with TaqMan Universal PCR Master Mix (Applied Biosystems) in 384-well plates in a final reaction volume of 10 μl. PCR was conducted using a 7900 HT Sequence Detection System (Applied Biosystems) and data were analyzed using SDS 2.3 software package (Applied Biosystems).

### Statistical Analysis

Class comparison was conducted with BRB Array Tools using a random variance model. Significant genes were defined as *p*-value < 0.001 and FDR < 0.1. Hierarchical cluster analysis and TreeView software were used for data visualization (Eisen Lab, http://rana.lbl.gov)[21]. Partek Genomic Suite 6.4 (Partek Inc., St. Louis, MO, USA) was used for the Principal Component Analysis. Database for Annotation, Visualization and Integrated Discovery (DAVID) 2008 software[22,23] was used for Gene Ontology (GO) enrichment analysis. For the analysis of specific pathways related to DC functions all the genes that, according to Biocarta (http://www.biocarta.com), are part of a specific pathway were selected. For each pathway, similarly to Chaussabel et al 2008[24] a less stringent *p-*value (0.05) and FDR (0.15) filter was applied and the remaining number of genes was arithmetically computed according to their up/down-regulation.

## Results

### GM-CSF and 15 kDa Granulysin induce partially overlapping monocyte signatures

**Figure 1:**
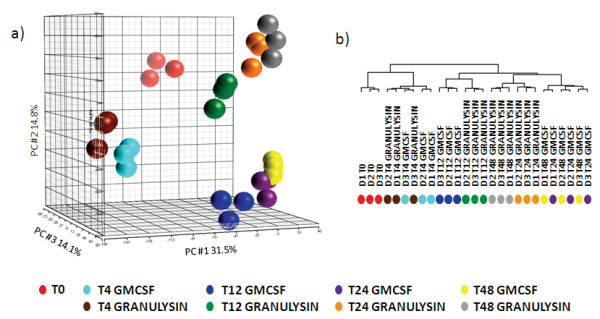
**Gene expression analysis of monocytes cultured with GM-CSF or 15 kDa Granulysin**. a) Principal component analysis of all samples based on the entire dataset (33757 genes); b) Dendrogram of the unsupervised cluster of 9951 genes that were present in at least 22 samples and whose expression differed in at least 5 samples by more than 1.75-fold from the median

Elutriated monocytes were cultured in presence of GM-CSF (10 ng/ml, 56 IU/ml) or 15 kDa Granulysin (10 nM). At 4, 12, 24 and 48 hours RNA was isolated and used for global gene expression analysis. Principal component analysis of the entire dataset (Figure 1a) revealed that GM-CSF and 15 kDa Granulysin induced a response in monocytes that was similar at early time points (4 hours) but strongly differed at later time points (12, 24 and 48 hours). In particular, principal component (PC) #1 which accounted for 31.5% of the variability of the dataset did not separate the samples cultured with Granulysin from those cultured with GM-CSF, but clearly placed the 4 and 48 hour samples at the extremes with the other samples in between and closer to the 48 hour samples. This indicated that the two agents induced one group of genes at 4 hours and a second set at later times. PC #2, which accounted for 14.8% of the variability, split the GM-CSF and Granulysin samples into two distinct groups at later time points, indicating that the differences between the GM-CSF- and Granulysin-cultured monocytes became more evident at later time points. The third PC (14.1% of the variability) segregated time 0 samples, the untreated monocytes, from the other samples indicating that both agents induced major changes at the transcriptome level when compared to time 0 samples.

In order to stratify changed transcripts associated with treatment and time in an unbiased fashion, the complete gene set was further filtered to include genes with expression levels ≥ 1.75-fold from the median in at least 20% of the samples[25]. 9951 out of 33757 genes were obtained and used for an unsupervised hierarchical cluster analysis which clearly separated early time point samples (T0 and T4) from the late time point samples (Figure 1b). Moreover, within the cluster of the late time point samples, three subclusters emerged: all 12-hour samples, the late 15 KDa Granulysin and late GM-CSF samples. This analysis revealed that GM-CSF and Granulysin induce in monocytes similar changes at the transcriptome level at early time points, but differences become more evident at later time points.

### GM-CSF and 15 kDa Granulysin induce the expression of several genes related to apoptosis and cell differentiation

**Figure 2:**
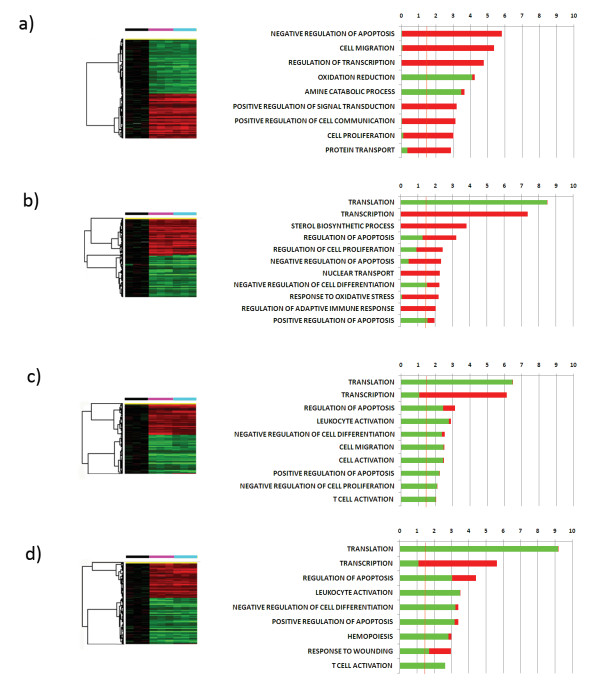
**Monocyte genes induced by both GM-CSF and 15 kDa granulysin**. Hierarchical clustering of the 3191, 2416, 1534 and 1738 genes induced by both factors at 4 (a), 12 (b), 24 (c) and 48 (d) hours respectively (p-value < 0.001, FDR < 0.1) and the related GO analysis. The hierarchical clustering was T0 corrected; the black bar indicates T0 monocytes, the fuchsia bar GM-CSF-treated monocytes, and the light blue bar Granulysin-treated monocytes. GO analyses were made with DAVID. The bars indicate -Log10 of the p-value of the overrepresentation of genes induced in each GO family. Green bars indicate down-regulated genes, while red bars indicate up-regulated genes. The orange line indicates the threshold of statistical significance (p-value = 0.05)

To analyze genes significantly induced by both GM-CSF and Granulysin compared to time 0 monocytes, we selected only the genes that at each time point were commonly induced following treatment by both agents compared to time 0 monocytes (t-test with p-value < 0.001 and FDR < 0.1). A total of 3191, 2416, 1534 and 1738 genes were induced by both GM-CSF and Granulysin at 4, 12, 24 and 48 hours respectively. We then evaluated gene ontology (GO) families that were statistically overrepresented among up- and down-regulated genes at each time point (Figure 2a, b, c, d). Genes related to apoptosis and cell differentiation were significantly enriched at almost all time points. In particular, genes that negatively regulate apoptosis were up-regulated at 4 hours, whereas at later time points those involved with positive induction of apoptosis were mainly down-regulated, suggesting a general down-regulation of apoptosis at each time point. The opposite was observed regarding proliferation related genes, with proliferation related genes mainly up-regulated at 4 hours and the negative regulation of proliferation related genes down-regulated at later time points, pointing to a general induction of cell proliferation. Moreover, at later time points, genes encoding zinc finger proteins were up-regulated and those encoding ribosomal proteins were down-regulated. Interestingly, at 12 hours both GM-CSF and Granulysin induced genes related to the regulation of the adaptive immune response, including CD40, CD80, PVR, PVRL2 and IDO1. This initial activation of the immune system was followed at 24 and 48 hours by the down-regulation of genes involved in leukocyte activation and proliferation, such as IL-8, IL-15, RAB27A, BCL11, FYN and CLCF1.

### The GM-CSF-specific gene expression signature

**Figure 3:**
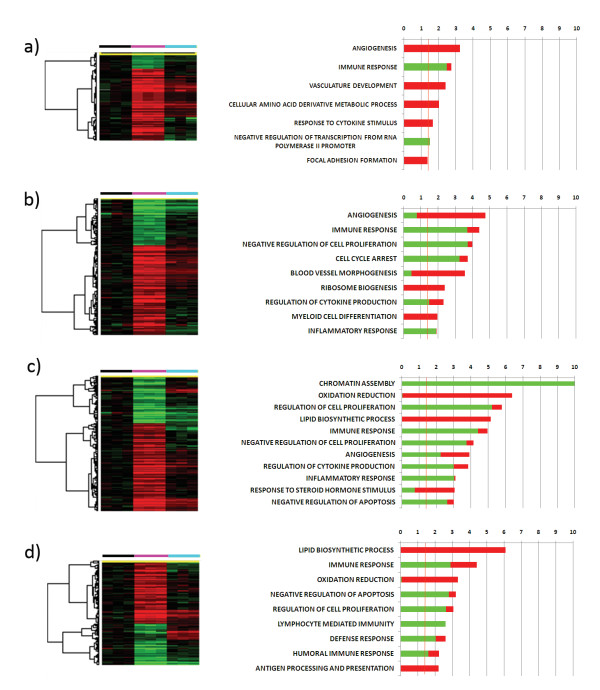
**Monocyte genes specifically induced by GM-CSF**. Hierarchical clustering of the 98, 768, 756 and 467 genes induced by GM-CSF at 4 (a), 12 (b), 24 (c) and 48 (d) hours respectively (p-value < 0.001, FDR < 0.1) and the related GO analysis. The expression of each of these genes differed in GM-CSF treated monocytes compared to both time 0 monocytes and cells treated with Granulysin at the same time points. The hierarchical clustering was T0 corrected; the black bar indicates T0 monocytes, the fuchsia bar GM-CSF-treated monocytes, and the light blue bar Granulysin-treated monocytes. GO analyses were made with DAVID. The bars indicate -Log10 of the p-value of the overrepresentation of induced genes in each GO family. The green bars indicate down-regulated genes, while the red bars indicate up-regulated genes. The orange line indicates the threshold of statistical significance (p-value = 0.05)

To identify genes specifically induced by GM-CSF at each time point we selected only the genes that were differentially expressed (*p*-value < 0.001 and FDR < 0.1) by GM-CSF-treated monocytes versus both time 0 monocytes and cells treated with Granulysin at the same time points. A total of 98, 768, 756 and 467 genes were specifically induced in GM-CSF-treated monocytes at 4, 12, 24 and 48 hours, respectively (Figure 3). Gene functional categories defined by Gene Ontology (GO) families at each time point were analyzed and only those overrepresented in both up- and down-regulated genes were illustrated (Figure 3). Interestingly, GM-CSF-treated monocytes specifically down-regulated immune related genes at each time point, among which were IL-10, CXCL1, CXCL2, CXCR4, CXCR5, and the co-stimulatory molecules CD27, CD28, FYB (ADAP) and TNFSF4 (OX40L). In particular, cytokine production, lymphocyte mediated immunity and humoral immune response GO families were overrepresented among the down-regulated genes at late time points. In contrast, at 48 hours, antigen processing and presentation were specifically up-regulated, including the overexpression of the genes CD1A, CD1B, CD1E, and HLA-DQA1. Moreover, at 12 hours, GM-CSF specifically up-regulated genes involved in myeloid cell differentiation, including IRF4, CSF1 (GCSF), RUNX1, CBFB and PPARG. In addition, at 12 hours, GM-CSF specifically induced the down-regulation of genes related to cell cycle arrest (among which were the cyclin-dependent kinase inhibitors CDKN1B, CDKN2B, CDNK1C), and thus favored cell proliferation. However, at 48 hours, anti-apoptotic genes, such as PIM3, THBS1, HGF and SERPINB2, were mainly down-regulated. Additionally, among the up-regulated genes specifically induced by GM-CSF at early time points were angiogenesis genes, while at late time points lipid biosynthetic process genes were up-regulated and several histone genes were down-regulated.

### The 15 kDa Granulysin-specific gene expression signature

**Figure 4:**
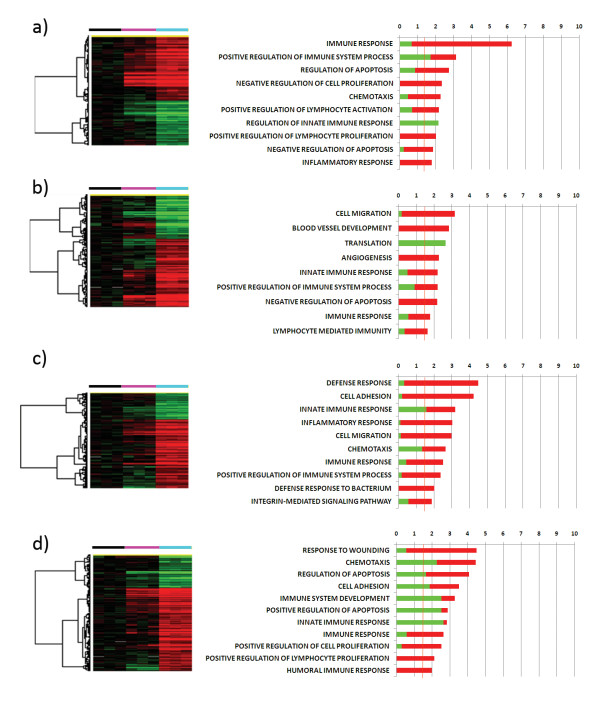
**Monocyte genes specifically induced by 15 kDa Granulysin**. Hierarchical clustering of the 152, 498, 429 and 598 genes induced by 15 kDa Granulysin at 4 (a), 12 (b), 24 (c) and 48 (d) hours respectively (p-value < 0.001, FDR < 0.1) and the relative GO analysis. The expression of each of these genes differed in Granulysin treated monocytes compared to both time 0 monocytes and cells treated with GM-CSF at the same time points. The hierarchical cluster analysis were T0 corrected, the black bar indicates T0 monocytes, the fuchsia bar GM-CSF-treated monocytes, and the light blue bar Granulysin-treated monocytes. GO analyses were made with DAVID. The bars in the GO analysis indicate -Log10 of the p-value of the overrepresentation of the induced genes for each GO family. The green bars indicate down-regulated genes, while red ones indicate up-regulated genes. The orange line indicates the threshold of statistical significance (p-value = 0.05)

A total of 152, 498, 429 and 598 genes were specifically induced in Granulysin treated monocytes at 4, 12, 24 and 48 hours, respectively, versus both time 0 monocytes and cells treated with GM-CSF at the same time points (*p*-value < 0.001 and FDR < 0.1, Figure 4). GO analysis showed immune response genes were up-regulated by Granulysin at each time point. In particular, a coordinated and time-dependent induction of immune related genes could be detected. At 12 hours, innate immunity related genes were up-regulated, but were later down-regulated. At 48 hours humoral and lymphocyte proliferative genes were mostly up-regulated, including CCL2, TNFRSF4, CD38, EBI3, C2, and C3. In addition, cell adhesion genes, including 4 integrins (ITGB8, ITGA9, ITGAV, ITGB5) and the chemokine C-C motif receptor 7 (CCR7), were specifically up-regulated especially at late time points. In addition, chemotaxis related genes were up-regulated at almost all time points, although the involved genes changed markedly between 4 and 48 hours. In fact, CXCL1, CXCL11, CCL20 and IL-6 were up-regulated at 4 hours, whereas CXCL3, CXCL12, CCL2, CCRL2, NRP2 and SEMA3A were induced at 48 hours. Of special note was the induction of cell proliferation genes: after the negative regulation of cell proliferation genes at 4 hours, a positive regulation of cell proliferation genes was most prominent at 48 hours.

### Granulysin, but not GM-CSF, activated pathways are related to DC function and common-host-response

**Figure 5:**
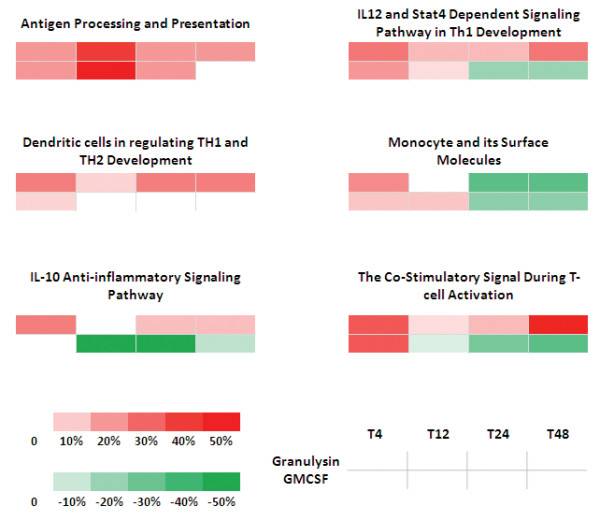
**DC related Biocarta pathway level analysis**. The percentages of genes statistically induced by each treatment and at each time point compared to time 0 monocytes are displayed in a grid (p-value < 0.05, FDR < 0.15). The position of each time-treatment in the grid is described in the bottom right corner, whereas the bottom left indicates the scale of intensity of the colors in the grid.

Since one of the main *in vitro *therapeutic uses of GM-CSF is the differentiation, in combination with IL-4, of monocytes into DCs and considering that our results suggest a partially similar response of monocytes when cultured with GM-CSF or 15 kDa Granulysin, we focused on 6 specific Biocarta pathways primarily involved in DC function (Figure 5). To evaluate the level of activation of each pathway we used gene lists with a less stringent cut off (*p*-value < 0.05 and FDR < 0.15)[24] and calculated the percentage of genes in each pathway induced by each treatment versus T0 monocytes. GM-CSF- and Granulysin-treated monocytes showed a similar number of genes in the Antigen Processing and Presentation, and Monocyte and Surface Molecules Pathways, although the former pathway revealed a constant up-regulation of genes, whereas for the latter pathway a down-regulation at late time points. In contrast, differences were observed regarding the other four pathways, reinforcing the observations described above. GM-CSF-treated monocytes clearly showed a unique down-regulation of the IL-10 Anti-Inflammatory Signaling and the Co-stimulatory Signal during T-cell Activation Pathways, whereas Granulysin-treated monocytes showed an up-regulation of genes in the latter pathway as well as those in the IL-12 and Stat4 Dependent Signaling in Th1 Development and Dendritic Cells in Regulating Th1 and Th2 Development Pathways. Almost the same conclusions could be outlined by focusing on the fold change of the genes in each pathway instead of the percentage of genes (data not shown).

To validate the microarray data, we performed real-time PCR on CCL2, CCR7, PIM1 and CD209 genes. The selection of CCL2 and CCR7 was based on their up-regulation in Granulysin-treated, but not GM-CSF-treated monocytes in array data. PIM1 was selected because it has been described as being induced by GM-CSF[26], and CD209 was selected since it is a marker of DC differentiation. The analysis was performed only on untreated T0 monocytes and hour 4 and 48 GM-CSF- and Granulysin-treated monocytes. Both CCL2 and CCR7 were statistically up-regulated by both agents at 4 hours, however, at 48 hours they were only up-regulated by Granulysin (*p*-value < 0.01) with a fold change greater than 70 for CCR7 and greater than 800 for CCL2 compared to time 0 monocytes, confirming the finding by microarray analysis (Additional file 1). At 4 hours the expression of both CCL2 and CCR7 was greater in Granulysin-treated monocytes than in monocytes treated with GM-CSF, with a fold change in Granulysin samples more than doubled for CCR7 and more than quadrupled for CCL2 compared to GM-CSF. Although PIM1 was filtered out in our analysis, RT-PCR showed a statistically significant induction of PIM1 at 48 hours (*p-*value < 0.05) by both GM-CSF and Granulysin, but its expression was greater in GM-CSF treated cells. This difference can be easily ascribed to the high stringency we used for statistical analysis of the microarray data (*p*-value < 0.001) where we preferred to select and analyze only those genes showing strong induction compared time 0 monocytes. In addition, we observed that CD209 was up-regulated by both agents at 48 hours (*p*-value < 0.01, with fold changes between 5 and 20 versus time 0 monocytes), which is similar to what we observed in the microarray dataset (both agents increased the expression CD209 genes with *p-*values < 0.0001).

## Discussion

GM-CSF has been used for immunotherapy both *in vivo *and *ex vivo *because of its stimulatory effect on immune system cells. Its main application for *ex vivo *immunotherapy is the differentiation of monocytes into DCs [9]. The broad utilization of GM-CSF in experimental conditions as well as in clinical use is partially due to the lack of alternative agents with similar activity. In this study, we performed a functional characterization of 15 kDa Granulysin side by side with GM-CSF and reported their impact on gene expression changes and kinetics in monocytes. Considering the stronger reliability of analyses of functional modules of genes compared to the analysis of single genes[24,27,28], we focused our analysis only on the pathways overrepresented among genes differently expressed with highly stringent *p*-values. Although it could be argued that several genes were not included in the analysis due to the high stringency, the use of these criteria ensured high sensitivity and specificity[29].

Our analysis showed that GM-CSF and 15 kDa Granulysin share similar functional property illustrated by their induction of large number of gene expression changes at different time points. The genes common to both agents were mainly related to cell differentiation and apoptosis; these genes enhanced the differentiation of monocytes and negatively impacted apoptosis. In addition, the common signature included immune response genes that were initially up-regulated in a similar fashion by both cytokines and were then down-regulated. However, beyond these overlapping functional characteristics, two different signatures specific to the agent were detected. The GM-CSF-specific signature revealed a down-regulation of immune response genes, among which were several co-stimulatory molecules. In contrast, Granulysin specifically and strongly induced genes related to the immune response with an initial activation on innate immune related genes followed by lymphocyte proliferative genes at later time points. In addition, cell adhesion genes were also specifically induced by Granulysin.

GM-CSF is a growth factor whose cellular effects had been studied for more than twenty years [30]. At low concentrations (< 1 pM) it induces only cell survival, but at higher concentrations it leads to monocyte proliferation, differentiation and functional activation[31]. We found that, although both GM-CSF and Granulysin induced genes related to cell differentiation and silenced genes related to cell death, only GM-CSF treated monocytes showed the down-regulation of cell cycle arrest genes, as previously described [31,32] and the up-regulation of genes involved in the myeloid cell differentiation. Moreover, our gene expression analysis not only confirmed the induction by GM-CSF of previously described genes, such as the anti-apoptotic gene IRF4[33], the proliferative gene PIM1[26], CSF1[34] and the macrophage inducer PPARG[35,36]; but also showed the up-regulation of the proliferation/differentiation regulator dimer RUNX1 -CBFB. RUNX1 -CBFB has not been previously reported to be up-regulated by GM-CSF and this observation merits further investigation.

Monocytes cultured in presence of GM-CSF alone are able to differentiate into iDCs, although these iDCs show a reduced ability to induce an effective activation of lymphocytes after maturation[36-38]. Our gene expression analysis clearly showed that GM-CSF leads to a specific down-regulation of several immune-related genes. Although we observed that GM-CSF induced a specific up-regulation of the well-known CD1 family genes [13], which play an important role in lipid antigen presentation; gene profiling also revealed a specific down-regulation of the co-stimulatory genes CD27, CD28, FYB (ADAP) and TNFSF4 (OX40L). Recent studies have shown how the proteins encoded by these genes are fundamental for the interaction of monocyte-derived dendritic cells and T and B cells [39-44]. In particular, GM-CSF derived DCs show a reduced ability to secret IL-12 after maturation [9,37]. Consistent with this, we observed a general specific down-regulation of the IL-12 and STAT4 Dependent Signaling Pathway in Th1 Development and the Co-stimulatory Signal during T-cell Activation Pathway. While these data suggest that GM-CSF treated monocytes might have a diminished ability to positively stimulate lymphocytes following antigen presentation, further focused functional studies are needed to test this hypothesis. Of particular interest is the observation that in the setting tested, GM-CSF specifically down-regulated IL-10, both the gene and the pathway, whereas previous results suggest that monocytes cultured in presence of GM-CSF produce high amounts of IL-10 once stimulated with LPS, IFN-γ, TNFα or anti-CD40 Ab [9,37]. This discrepancy could be the result of the differences in the concentration of GM-CSF used in the monocyte culture conditions or it may be that the higher expression of IL-10 by GM-CSF cultured monocytes is only subsequent to the stimulation with maturating agents.

15 kDa Granulysin is constitutively secreted in vivo by CTL and NK cells, but its function is still incompletely defined [15,17]. The ability of Granulysin to replicate some GM-CSF-induced monocyte responses is shown by the observation that between 4 and 48 hours thousands of genes were induced by both GM-CSF- and Granulysin. On the other hand, the gene expression analysis revealed that Granulysin, but not GM-CSF, treated monocytes showed an overexpression of several immune-related genes at each time point. Moreover, our data showed that Granulysin induced a specific time-coordinated activation of the immune system. At early time points, several genes involved in the activation of the innate immune system were induced whereas, at later time points, lymphocyte proliferation genes and humoral immune response were up-regulated. In addition, the pathway analysis clearly demonstrated that Granulysin-treated monocytes specifically induced the IL-12 and Stat4 Dependent Signaling Pathway in Th1 Development, suggesting that Granulysin might induce a shift towards Th1 T cell differentiation.

Recently, co-stimulatory molecules have been shown to play a role in chemotaxis [45]. We found that, in contrast to GM-CSF-treatment, Granulysin treatment did not lead to the down-regulation of co-stimulatory molecules; rather Granulysin specifically showed an up-regulation of the co-stimulatory pathways and overexpressed chemotactic genes at each time point. In particular, Granulysin induced the expression of a wide group of chemokines that are able to attract neutrophils (CXCL1, CXCL3)[46], memory and activated T cells (CXCL11, CCL20, CCR7)[47,48], monocytes (CCL2, CCL20)[49], macrophages and dendritic cells (NRP2)[50]. Several studies have shown that chemokines act synergistically[51,52], strengthening their signals and overcoming eventual antagonists secreted by pathogens[53,54]. Interestingly a partially overlapping time-fashioned chemokine induction has been described by myeloid and plasmacytoid DCs exposed to influenza virus[55]. This observation might indicate that 15 kDa Granulysin plays an important role in activating the immune system in response to pathogens by inducing monocytes to recruit other immune cells. Moreover, the observation that Granulysin acts as an alarmin strengthen this hypothesis [16,18].

## Conclusions

In conclusion, the analysis of gene expression profiles of monocytes cultured in presence of GM-CSF and 15 kDa Granulysin revealed that although both induce many of the same genes, these two cytokines induce two different monocyte responses. Considering the greater induction of several immune related functions by 15 kDa Granulysin, this study suggests that 15 kDa Granulysin may prove a useful therapeutic immunomodulator for *in vitro *production of Th-1 polarized monocyte-derived DCs for adoptive immunotherapy.

## Competing interests

AMK and CC hold patents on granulysin. The remaining authors declare no competing interests.

## Authors' contributions

LC performed experiments and data analysis; MWF expressed and purified the 15 kDa Granulsyin; DFS, MS, FMM, EW, CC, AMK contributed to experimental design and data analysis; LC, DFS compiled the manuscript; MS, FMM, EW, CC, AMK revised the manuscript. All of the authors have read and approved the final manuscript.

## Supplementary Material

Additional file 1 **Quantitative real time PCR analysis of selected genes**. Relative quantification of CCR7, CCL2, PIM1 and CD209 genes are represented. HPRT1 was used as a housekeeping gene. One sample of time 0 monocytes was set to the unitary value (1) and used as calibrator. Values from the 3 different donors were averaged and the standard deviation is represented for each bar. The light blue columns represent GM-CSF-treated monocytes and the purple bar Granulysin-treated monocytes. Click here for file
