# Supplementary figures and images for: 15 kDa Granulysin versus GM-CSF for monocytes differentiation: analogies and differences at the transcriptome level
Source: J Transl Med. 2011 Apr 18;9:41. doi: 10.1186/1479-5876-9-41 (PMC3094223; doi:10.1186/1479-5876-9-41)

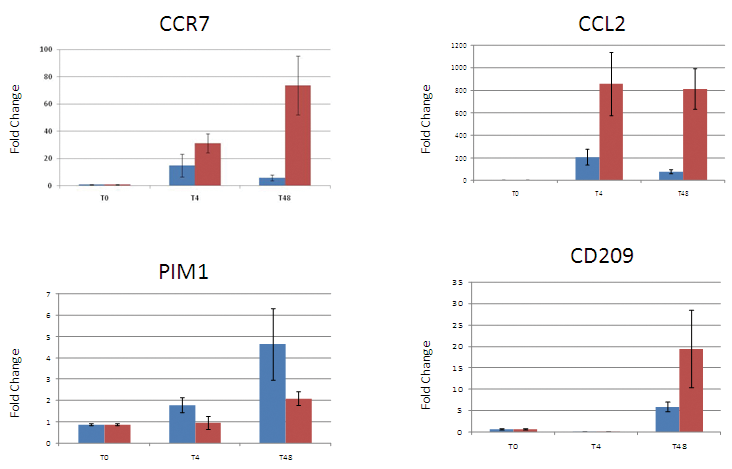

Supplement: Additional file 1 — Quantitative real time PCR analysis of selected genes. Relative quantification of CCR7, CCL2, PIM1 and CD209 genes are represented. HPRT1 was used as a housekeeping gene. One sample of time 0 monocytes was set to the unitary value (1) and used as calibrator. Values from the 3 different donors were averaged and the standard deviation is represented for each bar. The light blue columns represent GM-CSF-treated monocytes and the purple bar Granulysin-treated monocytes. [file 1479-5876-9-41-S1.TIFF]
